# Anti-Neuroinflammatory Effects of the Human Milk Oligosaccharide, 2′-Fucosyllactose, Exerted via Modulation of M2 Microglial Activation in a Mouse Model of Ischemia–Reperfusion Injury

**DOI:** 10.3390/antiox12061281

**Published:** 2023-06-15

**Authors:** Malk Eun Pak, Yeon-Ji Kim, Hanhae Kim, Chul Soo Shin, Jong-Won Yoon, Seon-min Jeon, Young-Ha Song, Kyungho Kim

**Affiliations:** 1 Korean Medicine-Application Center, Korea Institute of Oriental Medicine, Daegu 41062, Republic of Korea; clear46@kiom.re.kr (M.E.P.); yjikim@kiom.re.kr (Y.-J.K.);; 2 Korean Convergence Medical Science, University of Science and Technology, Daejeon 34054, Republic of Korea; 3 Advanced Protein Technologies Corp., Suwon 16229, Republic of Korea; csshin@aptech.biz (C.S.S.); jwyoon@aptech.biz (J.-W.Y.); smjeon@aptech.biz (S.-m.J.); yhsong@aptech.biz (Y.-H.S.)

**Keywords:** 2′-fucosyllactose, microglia polarization, neuroinflammation, reactive oxygen species, ischemic injury

## Abstract

Cerebral ischemic stroke is one of the leading causes of death and disability worldwide. 2′-fucosyllactose (2′-FL), a human milk oligosaccharide, exerts anti-inflammatory effects and plays a protective role in arterial thrombosis; however, its role in ischemic stroke remains unclear. This study aimed to investigate the neuroprotective effects of 2′-FL and its potential mechanisms in a mouse model of ischemic stroke. Neurological score and behavior tests revealed that 2′-FL promoted the recovery of neurological deficits and motor function in middle cerebral artery occlusion (MCAO) mice, and that 2′FL led to a reduction in the size of cerebral infarct. Biochemical studies showed that administration of 2′-FL led to a reduction of reactive oxygen species (ROS)-related products in the brain of MCAO mice. 2′-FL upregulated IL-10 and downregulated TNF-α level. In addition, 2′-FL enhanced M2-type microglial polarization and upregulated CD206 expression at 7 days after MCAO. At 3 days after MCAO, 2′-FL increased IL-4 levels and activated STAT6. Our data show that 2′-FL reduced the neurological symptoms of ischemic stroke and ROS accumulation in the brain through IL-4/STAT6-dependent M2-type microglial polarization in MCAO mice. These results demonstrate that 2′-FL is a potentially effective therapeutic agent for ischemic stroke.

## 1. Introduction

Cerebral ischemic stroke is one of the leading causes of disability and mortality worldwide and represents a significant health burden in both developed and developing countries [1]. The initial injury in a stroke occurs within minutes when blood flow to the injured focal core of the brain is limited, resulting in a significant reduction in oxygen and glucose delivery to neurons [2]. The ischemia–reperfusion injury process accelerates neuronal cell death due to energy depletion, inducing various postischemic responses that exacerbate brain damage and trigger pathological events. These include an increase in reactive oxygen species (ROS), disruption of the blood–brain barrier (BBB), activation of apo-necrotic cell death, excitotoxicity, and excessive production of inflammatory mediators [3]. The brain responds to neuronal damage caused by ischemia–reperfusion injury by activating various anti-inflammatory responses to confer neuroprotection. Accordingly, the use of anti-inflammatory agents may be a promising therapeutic strategy for cerebral ischemic injury [4,5].

Glial cells, particularly microglia, play a major role in the immunological response following an ischemic stroke [6]. Microglia undergo rapid morphological changes, polarizing into pro- or anti-inflammatory phenotypes and thus steering the course of degeneration or eventual recovery following stroke injury [7]. M1 activation produces the inflammatory cytokines tumor necrosis factor-alpha (TNF-α) and interleukin (IL)-6, as well as ROS. M2 activation produces the anti-inflammatory cytokines IL-10 and transforming growth factor (TGF)-β, along with other growth factors [8,9]. Microglial polarization and modulation of microglial phenotypes from M1 to M2 have been found to promote brain repair [10] and treat ischemic stroke [11].

Human breast milk is an excellent source of nutrients and contains various essential bioactive ingredients, such as human milk oligosaccharides (HMOs), which are its third most abundant solid component [11]. Previous studies have reported that breastfeeding not only provides complete nutrition to infants in the first months of life, protects them from infectious diseases, and necrotizes enterocolitis in newborns, but also has good effects on adult health [12]. Fucosyllactose is a major constituent of HMOs, and occurs as Fuc(α1-2)Gal(β1-4)Glc, also known as 2′-FL, which is the most abundant HMO in human milk, accounting for 20% of total HMOs [13]. 2′-FL is similar to the H-antigen of the blood group found on glycoproteins and glycolipids [14]. Infant formula containing 2′-FL and lacto-N-neotetraose (LNnT) has been reported to support age-appropriate growth, as well as lower rates of morbidity and medication use [15]. Adults supplemented with 2′-FL and LNnT displayed increased levels of Bifidobacterium with no side effects, indicating that HMOs have potential in providing nutrition beyond infant formula [16]. Preclinical studies have shown that HMOs, particularly 2′-FL, are more than a prebiotic and have various functions, including leukocyte adhesion, host–microbe interactions, and neural development [17,18,19,20,21,22,23,24]. Thus, the abundance of 2′-FL in human milk suggests a selective pressure with regard to the development of beneficial effects on infant physiology. Although there is increased evidence on the promising effects of 2′-FL, its neuroprotective effects have not been demonstrated in ischemia–reperfusion injury. Thus, the present study aimed to investigate the protective effects of 2′-FL against ischemic stroke via the modulation of microglial polarization. This study showed that 2′’-FL led to a reduction in the size of cerebral infarct and ROS-related products in the brain of mice with middle cerebral artery occlusion (MCAO) mice. Using biochemical approaches, we revealed that 2′-FL enhanced M2-type microglial polarization at 7 days after MCAO, and increased IL-4 levels and caused STAT6 activation at 3 days after MCAO. Hence, 2′-FL can serve as an effective therapeutic agent for the treatment of ischemic stroke.

## 2. Materials and Methods

### 2.1. Reagents

2′-FL was obtained from Advanced Protein Technologies Corp. (Suwon, Republic of Korea). The antibodies against β-actin, NQO1, STAT6, and phospho-STAT6 (pSTAT6) were obtained from Santa Cruz Biotechnology (Dallas, TX, USA) and the antibody against PGC1-α was obtained from Novus Biologicals (Centennial, CO, USA). The antibodies against inducible nitric oxide synthase (iNOS) and phospho-AMPKα (pAMPKα) were obtained from Cell Signaling (Danvers, MA, USA), and the antibodies against CD206 and Iba1 were obtained from Abcam (Cambridge, UK). The antibody against-CD16/32, enzyme-linked immunosorbent assay (ELISA) kit for interleukin (IL)-10, IL-4, and TNF-α, H_2_DCFDA, and radioimmunoprecipitation assay (RIPA) lysis buffer were obtained from Thermo Fisher (Waltham, MA, USA). Horseradish peroxidase (HRP)-conjugated secondary antibodies were obtained from Bethyl (Farmingdale, NY, USA), and enhanced chemiluminescence (ECL) solution was purchased from Pierce (Rockford, IL, USA). ELISA kits for catalase and superoxide dismutase (SOD) were obtained from Enzo Life Sciences, Inc. (Farmingdale, NY, USA).

### 2.2. Animals

Male mice (C57BL/6, 6 weeks old, body weight [BW] 17–18 g) were obtained from DooYeol Biotech (Seoul, Republic of Korea) and housed under constant conditions of temperature (21 °C ± 2 °C) and humidity with a 12 h light/dark cycle. The Institute of Animal Care and Use Committee of the Korea Institute of Oriental Medicine approved all experiments, which were performed according to their guidelines (approval number. KIOM-D-20-077).

### 2.3. Drug Administration

2′-FL dissolved at a concentration of 1 g/kg (BW) in a volume of 0.2 cc of water. It was administered orally prior to surgery, and next day after MCAO. Only water was administered orally to the control, sham, and vehicle groups. A focal cerebral ischemic injury was induced in mice in the vehicle and 2′-FL groups through surgery.

Experiment (I): Overall, 27 mice were randomly divided as follows: (1) sham group (no surgery, n = 3); (2) vehicle group (surgery, n = 3) administered with water at 1, 3, 7, and 14 days after surgery; and (3) 2′-FL group (surgery, n = 3) administered with 2′-FL at 1, 3, 7, and 14 days after surgery. 2′-FL was administered orally for 7 days prior to surgery and for 1, 3, 7, or 14 days from the first day after MCAO. Vehicle groups received only water (administered orally).

Experiment (II): Mice were then divided randomly into 5 groups of 38 animals each: (1) control group (no surgery, n = 6) administered with water; (2, 3) vehicle group (surgery, n = 16) administered with water at 3 and 7 days after surgery; and (4, 5) 2′-FL group (surgery, n = 16) administered with 2′-FL at 3 and 7 days after surgery. 2′-FL (1 g/kg BW) was administered orally for 7 days prior to surgery and for 3 or 7 days from the first day after MCAO.

### 2.4. Middle Cerebral Artery Occlusion (MCAO) Model

To induce focal cerebral ischemia, an MCAO model was established using the intraluminal filament technique. An MCAO model was induced by the insertion of the common carotid artery (CCA) according to the Longa’s method [25]. Mice were anesthetized with 2.0% isoflurane to minimize their suffering and cerebral blood flow was measured by attaching a probe for Laser Doppler (ADI Instruments, Dunedin, New Zealand) to the mouse skull. Mice were then placed in the supine position on a heating pad. After shaving on the neck region and disinfecting the surgical site, we created a 1 cm long midline incision. Then, the right CCA, external carotid artery (ECA), and internal carotid artery (ICA) were dissected carefully. The ECA and lower CCA were tied with sutures and a vascular clamp mediated via to the bifurcation of the CCA into the ECA and ICA. After making a small incision at the upper CCA, a silicon-coated 7-0 monofilament (L.M.S. Korea Inc., Gyeonggi, Republic of Korea) was inserted through an incision to ICA until MCA was occluded. After 45 min, the inserted filament was withdrawn, followed by reperfusion. The mice were kept warm to maintain their body temperature at 37 °C throughout the procedure. Subsequently, they were monitored until they woke up from anesthesia, after which they were returned to their cages. The survival rate of this MCAO surgery was approximately 85%.

### 2.5. Behavioral Experiments

#### 2.5.1. Neurological Function Evaluation

Twenty-four hours after ischemic injury, neurological deficits were evaluated on a five-point scale [26] as follows: 0—no deficit; 1—failure to fully extend left forepaw; 2—resistance to contralateral pressure without turning and with forepaw buckling; 3—resistance to lateral pressure with left turning and forepaw flexion; and 4—no spontaneous walking, with depressed level of consciousness. The inclusion criterion of the model was a neurological function score of 1–3, and the exclusion criterion was a neurological function score of 0 or 4.

#### 2.5.2. Corner Test

To test for sensorimotor dysfunction, we performed the corner test on days 3 and 7 after MCAO surgery. Mice suffering from MCAO-induced brain damage have functional deficits on the contralateral side [27] and therefore use their ipsilateral paws more often when turning corners. To assess this, two boards (30 cm × 20 cm) were attached at an angle of 30°, with one side left open. The direction in which the body of the mouse was raised at the inside corner was assessed. This procedure was repeated 10 times for each animal, and its number of turns to the ipsilateral side was calculated as a percentage.

#### 2.5.3. Wire Grip Test

The wire grip test was performed on days 3 and 7 after MCAO surgery to monitor motor balance and grip strength. Mice were suspended, allowing them to hold on to a single wire 50 cm above the ground [28]. Performance was measured as follows: 0—not able to grip the wire; 1—hangs on to wire with one or two forepaws; 2—attempts to climb on the wire while holding it with a forepaw; 3—grasps the wire with one or both hind paws, in addition to forepaws; 4—wraps the tail around the wire while holding it with all four paws; and 5—reaches one end of the wire and escapes from the apparatus. Time to fall off the wire was also scored: 0—unable to hold on, falls in under 1 s; 1—holds for 1–9 s; 2—holds for 10–19 s; 3—holds for 20–29 s; and 4—holds wire for >30 s. The total score was presented as the sum of the performance and hanging time scores.

### 2.6. Reverse Transcription–Polymerase Chain Reaction (RT-PCR)

Brain tissue from the ipsilateral side at 1, 3, 7, and 14 days after MCAO was homogenized in TRizol (Invitrogen), and total RNA was extracted using RNeasy Mini RNA isolation kit (Qiagen, Chatsworth, CA, USA) according to the manufacturer’s instructions. Then, 1 µg of RNA was used to synthesize cDNA using Omniscript Reverse Transcriptase (Qiagen). SYBR green-based quantitative PCR amplification was performed using the QuantStudio 6 Flex Real-time PCR System (Thermo Fisher Scientific, Waltham, MA, USA). The expression of RNA was calculated with the 2^−ΔΔCt^ method and normalized by GAPDH expression level. Results are shown in comparison to the sham group (no surgery). Primers used to amplify the genes of interest are listed in Table 1.

### 2.7. Nissl Staining

Mouse brains were removed, and tissue was fixed and frozen in an optimal cutting temperature compound. Subsequently, 30 μm thick sections were cut and mounted on slides. To evaluate brain edema, the sections were stained with 0.1% cresyl violet for 5 min. The sizes of subareas including the striatum, cortex, and hippocampus on both contralateral and ipsilateral sides were measured using ImageJ 1.52v software. Brain damage volume was expressed as a percentage: atrophy volume = subarea volume of the ipsilateral hemisphere/total volume of brain × 100.

### 2.8. Enzyme-Linked Immunoassay (ELISA)

Whole brain tissue, including both the contralateral and ipsilateral sides, was homogenized with RIPA lysis buffer and incubated for 20 min. Samples were spun down at 12,000 rpm at 4 °C for 20 min and the supernatant was collected. Next, 1 ug protein was calculated and evaluated for catalase (ADI-907-027), SOD (ADI-SOD-157), IL-10 (#88-7105-88), TNF-α (#88-7323-24), and IL-4 (#BMS613) levels using ELISA kits according to the manufacturer’s protocol.

### 2.9. Western Blot

Total brain tissue was homogenized in RIPA lysis buffer containing protease and phosphatase inhibitors. An equal amount of protein (20 μg) was loaded into each well of a sodium dodecyl sulfate–polyacrylamide gel electrophoresis gel and then transferred to a PVDF polyvinylidene fluoride membrane. The membranes were blocked and incubated at 4 °C overnight with the following primary antibodies: anti-β-actin (1:1000, sc-47778), NQO1 (1:1000, sc-376023), HO-1 (1:1000, #82206, 1:1000), iNOS (1:1000, #2982), Iba1 (1:1000, ab178846), CD16/32 (1:1000, #14-0161-82), CD206 (1:1000, ab64693), pAMPKα (1:1000, #2531), PGC1-α (1:1000, NBP1-04676), pSTAT6 (1:1000, sc-136019), and STAT6 (1:1000, sc-374021). The next day, the membranes were washed and incubated with HRP-conjugated antibodies (A90-137P or A120-108P, 1:4000) for 1 h and developed using an ECL solution. Immunoreactivity was recorded using a digital imaging system (Q9 Alliance; UVITEC Ltd., England, UK). The results were quantified using ImageJ 1.52v software and normalized to β-actin.

### 2.10. Detection of ROS

Frozen brain sections were incubated with a blocking buffer for 1 h. Sections were incubated with 20 μM H_2_DCF solution in phosphate-buffered saline (PBS) for 15 min at room temperature on an orbital shaker. Then, they were washed in PBS three times for 5 min and mounted immediately onto the mounting medium (Vector Laboratories, Inc., Burlingame, CA, USA). Images were captured using a fluorescence microscope (Olympus, Tokyo, Japan). Green fluorescence density was measured using ImageJ 1.52v software, and results were normalized to those of the control group.

### 2.11. Immunofluorescence (IF) Staining

Frozen sections were incubated with a blocking buffer for 1 h. Sections were incubated with primary antibodies overnight in a dilution buffer at 4 °C. The following antibodies were used: Iba1 (1:500, #019-19741), CD206 (1:500), and pSTAT6 (1:500). The sections were then incubated with a fluorescent secondary antibody (Vector Laboratories, Inc., Burlingame, CA, USA) for 2 h in the dark. After mounting tissue on slides with a mounting medium (Vector Laboratories, Inc.), images were captured using a fluorescence microscope (Lion Heart FX; Agilent, Santa Clara, CA, USA). Iba1 density was measured using ImageJ 1.52v software, and CD206^+^ or pSTAT6^+^/Iba1^+^ cells in the infarction region on the ipsilateral side (penumbra) were counted.

### 2.12. Statistical Analysis

Data analysis was performed using GraphPad Prism 5. Statistical significance was assessed using Bonferroni’s test (one-way analysis of variance) for comparing multiple groups. A *p*-value of <0.05 was considered to indicate statistical significance.

## 3. Results

### 3.1. 2′-FL Modulates the Polarization of Microglia in Ischemic Stroke

**Figure 1:**
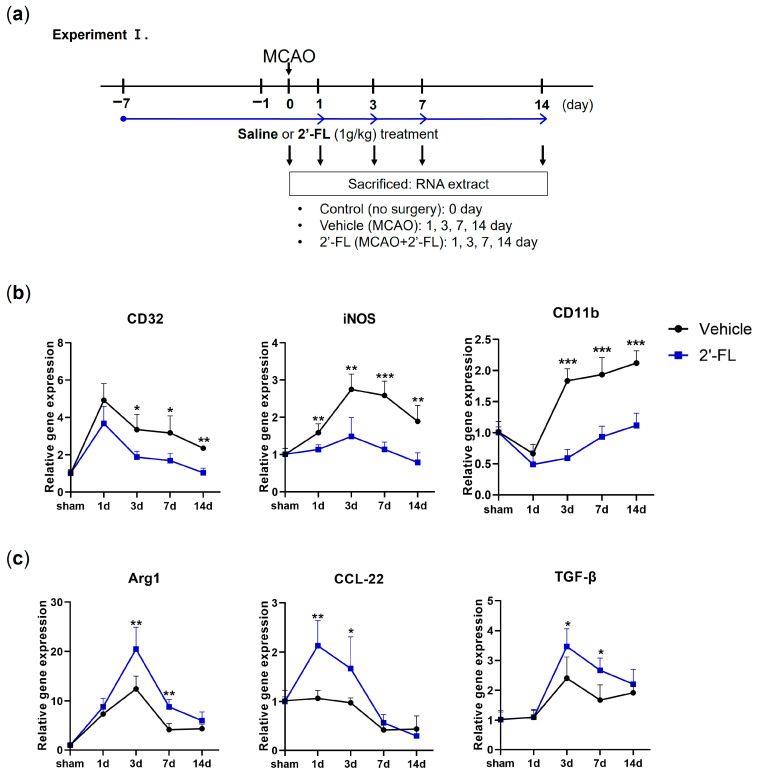
2′-FL induces a change in mRNA expression of M1 and M2 polarization markers in ischemic stroke. (**a**) Schedule for checking change of mRNA expression by 2′-FL treatment in middle cerebral artery occlusion (MCAO) brain (n = 3). (**b**) Expression of mRNA for M1 markers (CD32, iNOS, and CD11b) and (**c**) for M2 markers (Arg1, CCL-22, and TGF-β). Data are expressed as Mean ± SEM. * < 0.05, ** < 0.01 and *** < 0.001 vs. the vehicle group.

A previous study showed that M1 microglia markers exhibited an increasing trend during 14 days after ischemic stroke, and M2 markers increased from day 1 to day 7 and decreased after that [10]. To investigate the possible modulation of M1/M2-type microglia by 2′-FL, we examined marker lists for genes associated with M1-type (CD32, iNOS, and CD11b) and M2-type (Arg1, CCL-22, and TGF-β) microglia and then quantified the expression of these genes in our model using RT-PCR (Figure 1). We found that the gene expression of the M1-type in MCAO mice increased on day 3 compared with that in 2′-FL-treated mice (Figure 1b). 2′-FL treatment increased M2-type gene expression for 1 to 7 days compared with that in untreated MCAO mice (Vehicle) (Figure 1c). These data indicated that 2-’FL increases the expression of M2-type genes and maintains M2 activation for 3 to 7 days after ischemic stroke.

### 3.2. 2′-FL Abrogates Neurological Deficits and Motor Function in Ischemic Stroke

**Figure 2:**
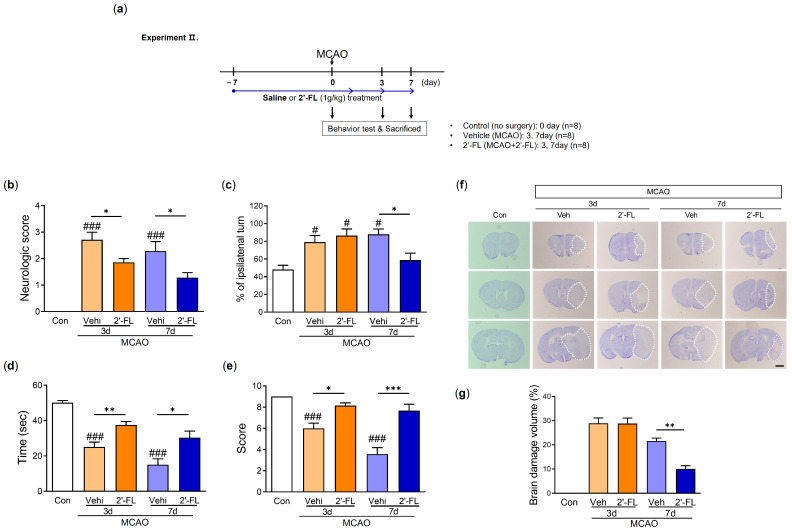
2′-FL improves neurological and motor function and reduced brain damage after ischemic brain injury. (**a**) Schedule of an experimental study. (**b**) Score of neurological deficits, (**c**) corner test, (**d**) time to fall down, and (**e**) score in wire grip test were evaluated 3 and 7 days after MCAO surgery (n = 7). (**f**) Nissl staining and (**g**) graph of brain tissue damage volume at 3 and 7 days after MCAO surgery (n = 4). A dot circle represents as brain infarction. Data are expressed as Mean ± SEM. # < 0.05, ### < 0.001 vs. the Con group, * < 0.05, ** < 0.01, *** < 0.001 vs. the vehicle group. Scale bar = 800 μm.

We next investigated how functional recovery in the mouse brain was affected by 2′-FL administration on days 3 and 7 after MCAO. Mice were given a neurological score and subjected to corner and wire grip tests to analyze their functional recovery (Figure 2a). The neurological score in the vehicle (MCAO) group on both days 3 and 7 post-MCAO markedly increased compared with that in the control group. 2′-FL improved neurological score on days 3 and 7 after MCAO (Figure 2b). In the corner test, mice in the vehicle group demonstrated significantly increased ipsilateral turning at 3 and 7 days post-MCAO, whereas 2-’FL treatment significantly reduced ipsilateral turning on day 7 (Figure 2c). In the wire grip test, the vehicle group showed a significantly reduced time to fall on both days 3 and 7 post-MCAO compared with the control group. However, the 2′-FL-treated group required a significantly longer time complete the wire grip test than the vehicle group on both days 3 and 7 (Figure 2d). The qualitative score via wire grip test in the vehicle group on day 7 was significantly reduced compared with control, and 2′-FL treatment improved score on day 7 post-MCAO (not significant on day 3) (Figure 2e). These data indicated that 2′-FL improves neurological and behavioral functions after ischemic stroke.

### 3.3. 2′-FL Reduces Neuronal Damage in the Brain of Ischemic Stroke

To investigate the effects of 2′-FL on histological changes in the brain after MCAO, we examined atrophy and damage in the frontal cortex, striatum, and hippocampus using Nissl staining. The neuronal damage was not observed in the brains of the control group, but MCAO mice treated with either vehicle or 2′-FL on day 3 after MCAO showed approximately 30% damage. We found that 2′-FL markedly reduced atrophy and damage in the brain compared with the vehicle group on day 7 (Figure 2f,g). These data indicated that the administration of 2′-FL reduced brain damage on day 7 after ischemic stroke.

### 3.4. 2′-FL Reduces ROS Generation in Ischemic Stroke

**Figure 3:**
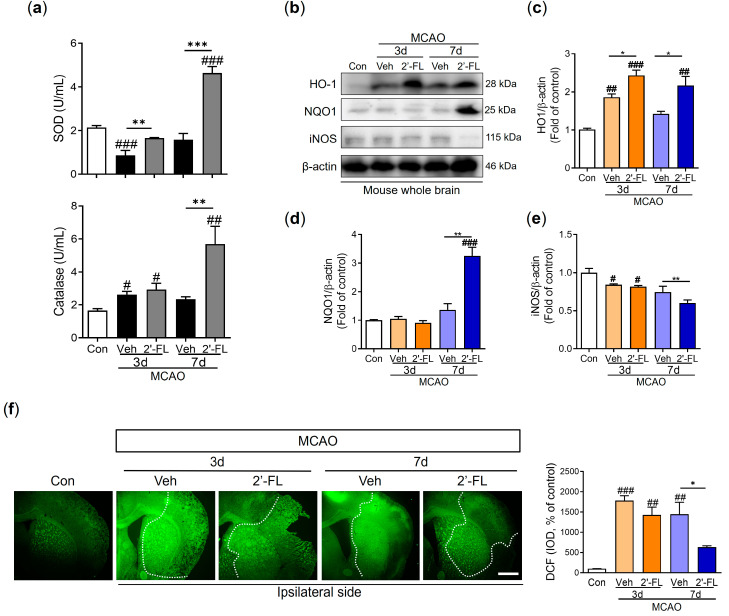
2′-FL attenuates ROS production after ischemic brain injury. Level of (**a**) SOD and catalase in the whole brain (n = 6). (**b**) Representative Western blot and its densitometric analysis of (**c**) HO−1, (**d**) NQO1, and (**e**) iNOS protein in whole brain at 3 and 7 days after MCAO (n = 3). (**f**) DCFDA staining and graph in intensity of density (IOD) of DCF at 3 and 7 days after MCAO surgery (n = 4). Data are expressed as Mean ± SEM. # < 0.01, ## < 0.05, ### < 0.001 vs. the Con group, * < 0.05, ** < 0.01, *** < 0.001 vs. the vehicle group. Scale bar = 400 μm.

To examine the effect of 2′-FL on ROS generation, we investigated the levels of SOD and catalase in the brain after MCAO. We observed that the level of SOD was reduced on day 3 after ischemic injury compared with the control group, and 2′-FL treatment increased these reduced levels. On day 7 after MCAO, the vehicle group tended to exhibit low SOD levels, and 2′-FL treatment markedly increased the levels compared with those in the control and vehicle. Further, the catalase level was induced by ischemic injury in the vehicle and 2′-FL groups on day 3. On day 7 after MCAO, 2′-FL treatment significantly increased catalase levels compared with those in the control and vehicle (Figure 3a). To further evaluate the modulation of antioxidant protein expression via 2′-FL treatment, we determined the expression levels of HO-1, NQO1, and iNOS using western blot analysis. Compared with the control group, we found increased levels of HO-1 and decreased expression levels of iNOS on day 3 after MCAO in both vehicle and 2′-FL-treated groups. On day 7, the 2′-FL-treated group showed markedly increased HO-1 expression and decreased iNOS expression compared with the vehicle group (Figure 3b–e). Interestingly, the expression of NQO1 was markedly increased in the 2′-FL-treated group on day 7 after MCAO, but not on day 3 (Figure 3d). We confirmed whether 2′-FL reduced ROS accumulation in brains with ischemic injury using DCFDA staining (Figure 3f). ROS accumulation in the brains of the vehicle group (ipsilateral side) increased compared with that in the control group on days 3 and 7; however, 2′-FL significantly decreased ROS accumulation on day 7. These data indicated that 2′-FL administration reduced ROS generation in the brain at 7 days after ischemic stroke.

### 3.5. 2′-FL Regulates Cytokines Related to Inflammation and Microglial Activation in Ischemic Stroke

**Figure 4:**
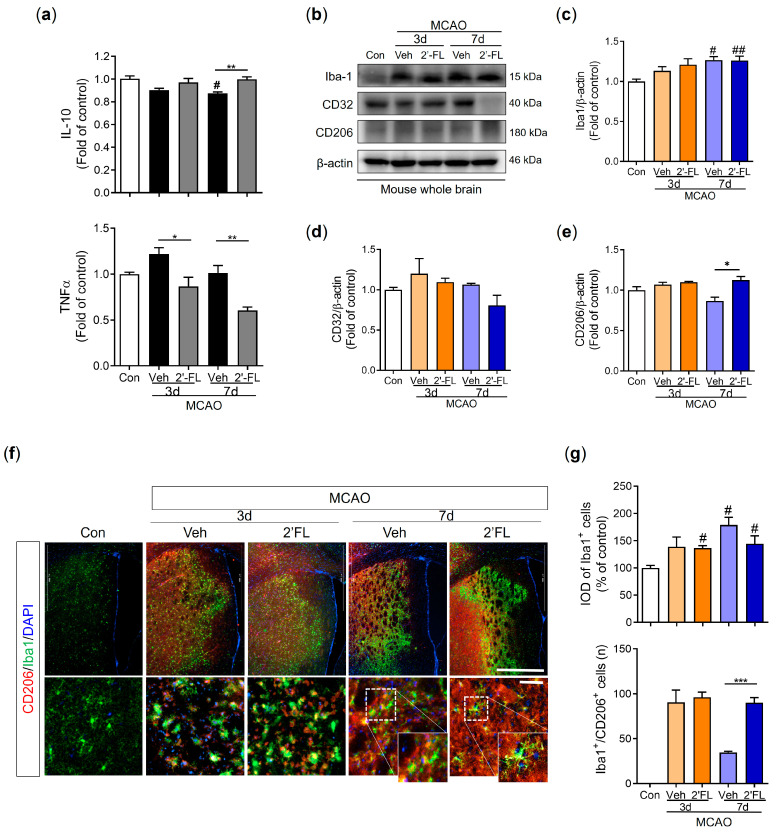
2′-FL activates microglia by regulating cytokines after ischemic brain injury. Level of (**a**) IL-10 and TNF-a in the whole brain (n = 6). (**c**) Representative Western blot and its densitometric analysis of (**b**) Iba1, (**d**) CD32, and (**e**) CD206 protein in the whole brain on 3 and 7 days after MCAO (n = 3). (**f**) Photomicrograph for CD206^+^/Iba1^+^ in the striatum of ipsilateral side and its histogram for (**g**) IOD of Iba1^+^ cells and the number of CD206^+^/Iba1^+^ cells (n = 3). Data are expressed as Mean ± SEM. # < 0.05, ## < 0.01 vs. the Con group, * < 0.05, ** < 0.01, *** < 0.001 vs. the vehicle group. Scale bar = 200 μm.

Because microglial polarization to the M2 type promotes brain repair and confers a beneficial effect after ischemic stroke [10,29], we investigated whether 2′-FL regulates IL-10 and TNF-α secretion using ELISA. IL-10 levels were decreased by MCAO compared to the control group, but 2′-FL-treated MCAO mice demonstrated significantly higher IL-10 levels than vehicle-treated MCAO mice on day 7. TNF-α levels in the vehicle group tended to increase compared to the control group on day 3 (not on day 7) and 2′-FL groups on both days 3 and 7 were reduced compared with those in the vehicle group (Figure 4a). Furthermore, we investigated the expression levels of Iba1, CD32, and CD206 proteins using Western blot analysis to confirm M1/M2 microglial polarization. The expression level of Iba1 on day 7 post-MCAO in the vehicle and 2′-FL groups was significantly increased compared with that in the control group, but there was no significant increase on day 3 (Figure 4b). We revealed that ischemic injury induced an increased CD32 expression level on day 3 compared with the control. On day 7, 2′-FL treatment tended to decrease the expression level of CD32 compared with that in the vehicle (Figure 4d). CD206 expression in the 2′-FL-treated group significantly increased compared with the vehicle group on day 7 after MCAO (Figure 4e). However, CD32 and CD 206 in the whole brain remained unchanged on day 3 (Figure 4d,e). To confirm whether 2′-FL activates M2-type microglia in the damaged brain region, we explored M2 activation in the infarct region (striatum and cortex on the ipsilateral side) after MCAO using IF staining (Figure 4f). We found that ischemic injury induced an increase in the intensity of density (IOD) of Iba1^+^ cells on the ipsilateral side. After 7 days, the IOD of Iba1^+^ cells in both vehicle and 2′-FL groups significantly increased compared with that in the control group. Compared with the control group, the number of Iba1^+^/CD206^+^ cells in the penumbra of both the vehicle and 2′-FL groups increased 3 days after MCAO; however, on day 7, the number of Iba1^+^/CD206^+^ cells in the 2′-FL-treated group was significantly higher than that in the vehicle group (Figure 4g). These data suggest that 2′-FL administration maintains M2 microglial activation for 7 days after ischemic stroke.

### 3.6. 2′-FL Activates M2 Type Microglia via IL-4 Induction and STAT6 Activation after MCAO

**Figure 5:**
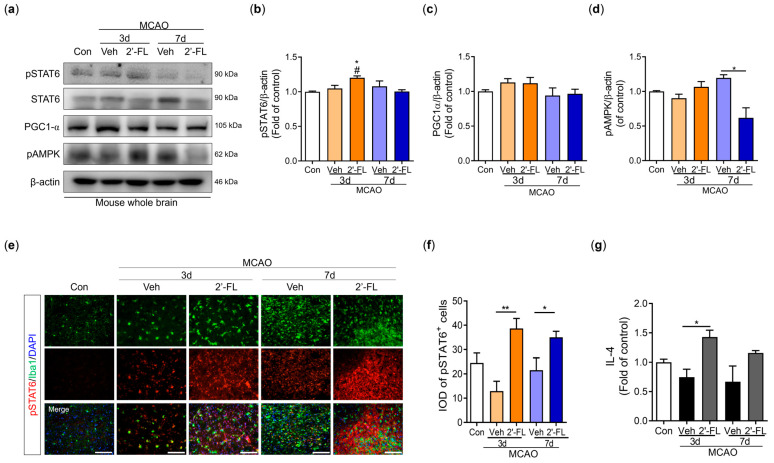
2′-FL activates STAT6 after ischemic brain injury. (**a**) Representative Western blot and its densitometric analysis of (**b**) pSTAT6, (**c**) PGC1α, and (**d**) pAMPK in whole brain at 3 and 7 days after MCAO (n = 3). (**e**) Photomicrograph for pSTAT6^+^/Iba1^+^ in the striatum of ipsilateral side and (**f**) its histogram for IOD of pSTAT6^+^ cells (n = 3). (**g**) Level of IL-4 in the whole brain (n = 6). Data are expressed as Mean ± SEM. # < 0.05 vs. the Con group, * < 0.05, ** < 0.01 vs. the vehicle group. Scale bar = 100 μm.

Microglial polarization to the M2 type is associated with the STAT6 pathway and PGC-1α expression. AMPK phosphorylates PGC-1α directly after ischemic injury [10,30]. We examined the expression of pSTAT6, PGC-1α, and pAMPK proteins. The phosphorylation level of STAT6 was increased in the 2′-FL-treated group compared with the control and vehicle groups on day 3 after MCAO but did not change on day 7 (Figure 5b). The expression of PGC-1α did not change among groups; however, the expression of AMPK was markedly decreased by 2′-FL on day 7 after MCAO (Figure 5c,d). To confirm pSTAT6 modulation by 2′-FL in the brain, we captured the fluorescence microscopic images of the ipsilateral striatum after MCAO. We found that the 2′-FL-treated group had a significantly increased number of Iba1^+^/pSTAT6^+^ cells compared with the vehicle group on the ipsilateral side (Figure 5e,f). IL-4 switches modulate the STAT6 pathway [31]; therefore, we assessed IL-4 levels in the brain using ELISA. We found that 2′-FL treatment significantly increased IL-4 levels on day 3 after MCAO compared with the vehicle group, but not on day 7 (Figure 5g). These data suggest that 2′-FL administration increased IL-4 levels and STAT6 phosphorylation on day 3 after ischemic stroke and led to a decrease in AMPK activation on day 7.

## 4. Discussion

Stroke is one of the leading causes of death and disability worldwide. The main cause of stroke is the occlusion or rupture of the brain’s blood vessels, which leads to oxygen deprivation in brain cells [32]. The progression of cerebral ischemic disease is greatly influenced by inflammation. Moreover, cerebral ischemia is accompanied by increased serum concentrations of oxidative stress markers [33]. Hence, anti-inflammatory and antioxidant agents are promising therapeutic targets for reducing secondary damage [34,35]. At present, the thrombolytic recombinant tissue plasminogen activator (rtPA) is the only FDA-approved therapy for acute ischemic stroke that can effectively diminish infarct size and improve functional recovery. However, rtPA has several disadvantages, including the potential risk of hemorrhagic transformation, a limited therapeutic time window after stroke onset, and limited efficacy. Consequently, it is still difficult to provide safe and effective therapies for ischemic stroke, especially at an early stage [36]. Therefore, developing and identifying therapeutic medications or alternatives that are beneficial for promoting functional recovery or quality of life following a stroke are necessary.

With an improved understanding of the mechanism underlying stroke, current research has focused on the duality of post-stroke inflammation, which is a significant contributing factor to the pathogenic process of stroke [37] and plays an important role in brain tissue damage and repair [38,39]. Enhancement of microglial activation is a potential therapeutic strategy for repairing ischemia, and studies on the modification of the microglial phenotype to promote healing processes may help identify new therapeutic targets [40,41]. In particular, M2 microglia produce anti-inflammatory cytokines, such as IL-10, thereby promoting cell debris phagocytosis, tissue repair, and neuron survival [41,42]. A previous study demonstrated that HMO treatment elevated the production of IL-10 and decreased the production of TNF-α in mouse mast cells, thereby alleviating the symptoms of food allergy [43]. In addition, our previous study showed that 2′-FL attenuates platelet activation and reduces stroke-induced infarct volume [44]. Based on these studies, we investigated whether 2′-FL could reduce ischemic-brain-injury-induced inflammation by increasing M2-type microglial activation. We found that 2′-FL significantly diminished brain infarct size and improved functional recovery. We also demonstrated that 2′-FL treatment significantly increased M2 microglial activation and expression of markers, such as CD206, in a mouse model of ischemic damage (Figure 4).

A previous study showed that an increase in the harmful accumulation of ROS caused by hypoxia-activated AMPK owing to an increase in the AMP/ATP ratio [45]. Similarly, we observed that 2′-FL treatment resulted in sustained M2 polarization after 3 days, which significantly decreased ROS formation. In turn, AMPK activation was markedly reduced at 7 days. Furthermore, the expression of the antioxidant proteins HO-1 and NQO-1 was substantially increased on days 3 and 7 after ischemic injury, whereas the expression of iNOS, a marker for M1 microglia, was not increased (Figure 3). Therefore, we anticipated that 2′-FL treatment would reduce M1 microglial activation at 7 days as a result of its continued support of M2 activation under ischemic conditions. Moreover, we found that 2′-FL induced STAT6 activation and IL-10 levels in the brain of mice with ischemic injury (Figure 4 and Figure 5). Several bioactive substances possess neuroprotective and anti-inflammatory properties, and most of these substances can alter the phenotype of microglia by activating them in a STAT6-dependent manner. For example, PGC-1α interacts with PPAR-γ, which is involved in IL-4-induced M2 polarization [9]. It has been demonstrated that resveratrol, a Sirt1 agonist, can modulate the microglia phenotype from M1 to M2 by upregulating PGC-1α expression and activating the STAT6 pathway. Furthermore, PGC-1α is directly phosphorylated by AMPK [30]. Interestingly, our findings demonstrated that 2′-FL only elevated phosphorylation of STAT6 in the early stage (3 days after ischemic injury), without associated changes in PGC-1α (Figure 5b,c), suggesting that STAT6 activation induced by 2′-FL treatment was followed by a decrease in AMPK activity.

**Figure 6:**
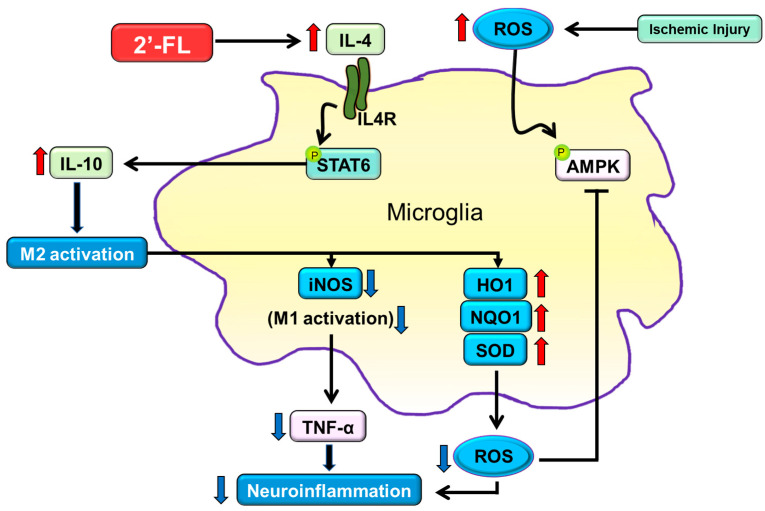
The proposed neuroinflammation mechanism of 2′-FL in regulating microglial M1/M2 polarization following ischemic injury via IL-4/STAT6 signaling pathways. Under ischemic injury conditions, STAT6 can be activated by IL-4 in the presence of 2′-FL, thereby regulating the expression of IL-10 and activating M2 microglial polarization. M2 activation leads to the inhibition of ROS generation through the downregulation of iNOS and the upregulation of HO1, NQO1, and SOD.

M2 macrophages are subclassified into M2a, M2b, and M2c. M2a contributes to the repair of damaged tissue by expressing anti-inflammatory and neurotrophic factors [46,47]. The M2a state can be induced by IL-4 and is associated with tissue repair and phagocytosis. IL-4 activates STAT6, thereby leading to the transcription of M2a-associated genes, including CD206. Interestingly, microglia cells of mice with ischemic injury showed large cell and soma areas with short branches [46]. However, 2′-FL treatment resulted in cells with a smaller soma area and higher branching (Figure 6). We, therefore, propose that 2′-FL treatment activates M2a microglia via IL-4–STAT6 signaling.

One limitation of our study is the inability to determine whether 2′-FL promotes IL-4 production directly or indirectly in the brain after ischemia. A previous study demonstrated that HMO treatment increased the levels of IL-10 and IL-6, but not TNF-α, in dendritic cells, which modulated the immune system via the promotion of T-cells [48]. This suggests that T-cells activated by 2′-FL may penetrate the brain and secrete IL-4. In addition, our data showed that 2′-FL upregulated antioxidant defenses such as SOD and HO-1 at an early time point after ischemic injury and later induced an increase in catalase and NQO-1 levels later (Figure 3e). These findings indicate that 2′-FL may have anti-inflammatory and antioxidant effects in the brain independent of IL-4 induction.

## 5. Conclusions

Our study showed that 2′-FL reduces ROS levels to protect against inflammation and oxidative stress caused by ischemic damage. Furthermore, treatment with 2′-FL maintained M2 microglial polarization through IL-4-induced STAT6 activation and IL-10 upregulation, which is crucial for the resolution of brain edema and recovery of motor behavior. Overall, these data suggest that 2′-FL may have the potential to be an effective therapeutic agent for patients with ischemic stroke. In addition, daily supplements of 2′-FL may serve as a viable preventive agent for ischemic injury.

## Figures and Tables

**Table 1:** Primers for RT-PCR. CD32, low-affinity immunoglobulin gamma Fc region receptor III-b; iNOS, inducible nitric oxide synthase; CD11b, integrin alpha-M; Arg-1, arginase-1; CCL-22, C-C motif chemokine ligand-22; TGF-β, transforming growth factor-β.

Gene	Primer (Forward)	Primer (Reverse)	Accession No.
CD32	AATCCTGCCGTTCCTACTGATC	GTGTCACCGTGTCTTCCTTGAG	BC038070
iNOS	CAAGCACCTTGGAAGAGGAG	AAGGCCAAACACAGCATACC	BC062378
CD11b	CCAAGACGATCTCAGCATCA	TTCTGGCTTGCTGAATCCTT	NM_008401.2
Arg1	TCACCTGAGCTTTGATGTCG	CTGAAAGGAGCCCTGTCTTG	NM_007482.3
CCL-22	CTGATGCAGGTCCCTATGGT	GCAGGATTTTGAGGTCCAGA	NM_009137.2
TGF-β	TGCGCTTGCAGAGATTAAAA	CGTCAAAAGACAGCCACTCA	NM_011577.2
GAPDH	TCAACAGCAACTCCCACTCTTCCA	ACCCTGTTGCTGTAGCCGTATTCA	GU214026.1

## Data Availability

All the data supporting the results were shown in the paper, and can be obtained from the corresponding author.
